# The Effect of the COVID-19 Pandemic on Suicide Attempts and Self-Harm in Teenagers and Young Adults: An Analysis of Regional Emergency Medical Center Data of a Metropolitan City in South Korea

**DOI:** 10.1192/j.eurpsy.2024.552

**Published:** 2024-08-27

**Authors:** S. M. Bae, N. Ku

**Affiliations:** ^1^psychiatry, Gachon University Gil Medical Center; ^2^College of Medicine, Gachon University, Incheon, Korea, Republic Of

## Abstract

**Introduction:**

The COVID-19 pandemic has had a wide-ranging impact on economic and social phenomena worldwide, particularly affecting mental health. However, these impacts have varied significantly across countries. Previous studies have shown that the groups more vulnerable to mental health problems also differ across countries and societies(Gunnel *et al*. Lancet Psy 2020; 7(6) 468-471, Pirkis *et al.* Lancet Psy 2021; 8(7) 579-588, Nomura *et al.* Psy Res 2021; 295 113622). Therefore, by examining changes in self-harm and suicide attempts, which constitute mental health emergencies, at the community level, we can explore the COVID-19 pandemic’s impact on the deterioration of mental health in various age groups within the region and identify the groups most vulnerable to mental health problems.

**Objectives:**

The aim of this study is to examine the relationship between the COVID-19 pandemic and changes in the number of emergency room visits due to suicide attempts or self-harm in teenagers and young adults in Incheon, a metropolitan city in the capital area of South Korea.

**Methods:**

We conducted a retrospective data analysis on the medical records of patients who visited the regional emergency center of Incheon Medical Institution from January 2018 to December 2022 due to suicide attempts or self-harm. As our statistical method, we employed interrupted time series analysis to determine whether the COVID-19 pandemic has a statistically significant correlation with the trend changes in the number of emergency room visits related to suicide attempts or self-harm. This study was approved by the Institutional Review Board of Gil Medical Center, Gachon University of Korea(IRB approval number GFIRB2022-335).

**Results:**

The data of 4,030 subjects(35.8% male; n=1,443) who visited the regional emergency center during the study period were analyzed. A total of 556 (13.79%) of the study participants were minors under the age of 19, and a total of 1,789 (44.39%) were young adults aged 20-39. The analysis revealed an increasing trend in the number of emergency room visits due to elevated suicidality in teenagers and young adults (20-39 years old) following the COVID-19 pandemic(figure 1 & figure 2).

**Image:**

**Figure:**
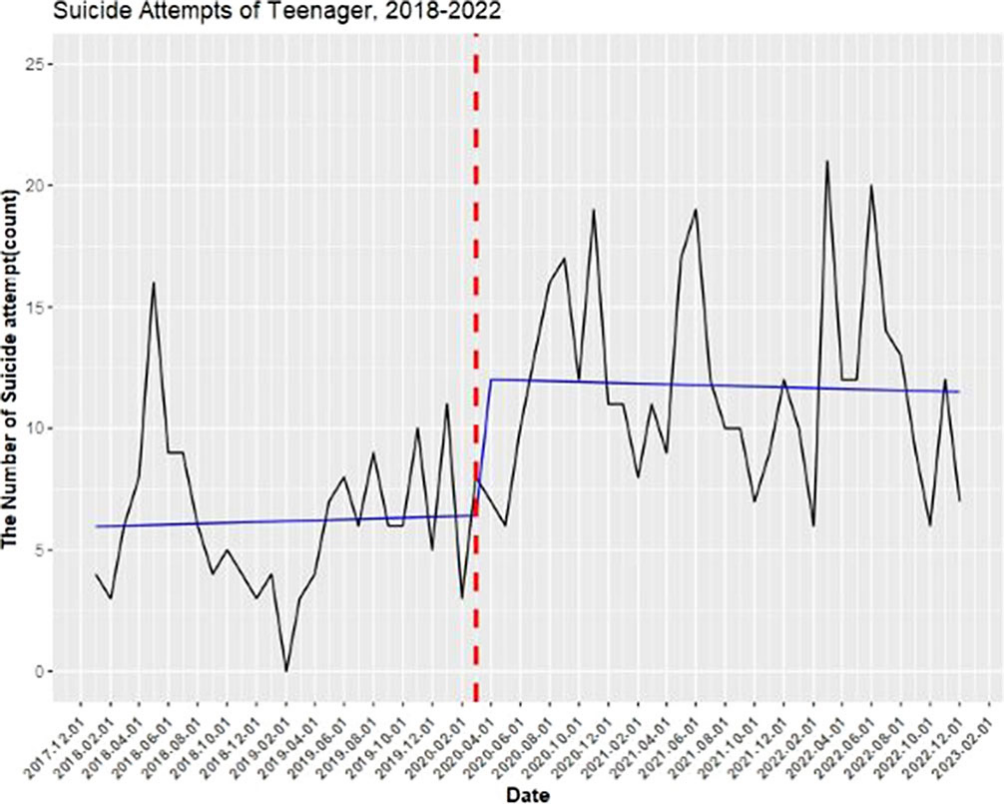

**Image 2:**

**Figure:**
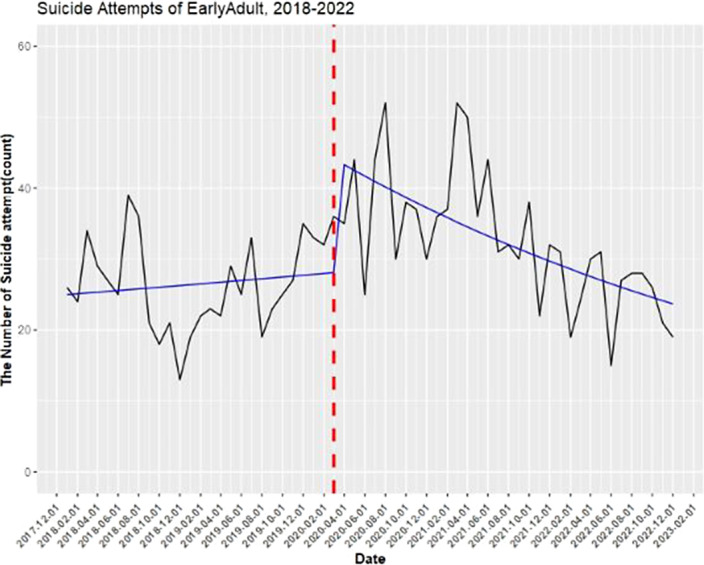

**Conclusions:**

While suicide attempts and visits to the emergency room due to self-harm increased both before and after COVID-19, it is noteworthy that past suicide attempts are the most significant risk factor for future suicide attempts. Therefore, the data on vulnerable groups presented in this study can be instrumental for effective prevention and follow-up management of suicide attempts within the field of community psychiatry.

**Disclosure of Interest:**

None Declared

